# Machine Learning Modelling and Feature Engineering in Seismology Experiment

**DOI:** 10.3390/s20154228

**Published:** 2020-07-29

**Authors:** Michail Nikolaevich Brykov, Ivan Petryshynets, Catalin Iulian Pruncu, Vasily Georgievich Efremenko, Danil Yurievich Pimenov, Khaled Giasin, Serhii Anatolievich Sylenko, Szymon Wojciechowski

**Affiliations:** 1 Zaporizhzhia Polytechnic National University, 69063 Zaporizhzhia, Ukraine; brykov@zntu.edu.ua (M.N.B.); dekanat_bad@zntu.edu.ua (S.A.S.); 2 Institute of Materials Research, Slovak Academy of Sciences, 04001 Kosice, Slovak; ipetryshynets@saske.sk; 3 Mechanical Engineering, Imperial College London, Exhibition Rd., London SW7 2AZ, UK; 4 Mechanical Engineering, School of Engineering, University of Birmingham, Birmingham B15 2TT, UK; 5 Pryazovskyi State Technical University, Physics department, 87555 Mariupol, Ukraine; vgefremenko@gmail.com; 6 Department of Automated Mechanical Engineering, South Ural State University, Lenin Prosp. 76, 454080 Chelyabinsk, Russia; danil_u@rambler.ru; 7 School of Mechanical and Design Engineering, University of Portsmouth, Portsmouth PO1 3DJ, UK; Khaled.Giasin@port.ac.uk; 8 Faculty of Mechanical Engineering, Poznan University of Technology, Piotrowo 3, 60–965 Poznan, Poland; sjwojciechowski@o2.pl

**Keywords:** seismology, earthquake prediction, laboratory experiment, acoustic data, machine learning, feature engineering, artificial intelligence

## Abstract

This article aims to discusses machine learning modelling using a dataset provided by the LANL (Los Alamos National Laboratory) earthquake prediction competition hosted by Kaggle. The data were obtained from a laboratory stick-slip friction experiment that mimics real earthquakes. Digitized acoustic signals were recorded against time to failure of a granular layer compressed between steel plates. In this work, machine learning was employed to develop models that could predict earthquakes. The aim is to highlight the importance and potential applicability of machine learning in seismology The XGBoost algorithm was used for modelling combined with 6-fold cross-validation and the mean absolute error (MAE) metric for model quality estimation. The backward feature elimination technique was used followed by the forward feature construction approach to find the best combination of features. The advantage of this feature engineering method is that it enables the best subset to be found from a relatively large set of features in a relatively short time. It was confirmed that the proper combination of statistical characteristics describing acoustic data can be used for effective prediction of time to failure. Additionally, statistical features based on the autocorrelation of acoustic data can also be used for further improvement of model quality. A total of 48 statistical features were considered. The best subset was determined as having 10 features. Its corresponding MAE was 1.913 s, which was stable to the third decimal point. The presented results can be used to develop artificial intelligence algorithms devoted to earthquake prediction.

## 1. Introduction

In recent years, artificial intelligence has been extensively used to solve problems in different fields of human or natural activities. Artificial intelligence methods are widely used in a variety of engineering applications, for example, in artificial intelligence-based hole quality prediction in micro-drilling [1], or in the prognosis of bearing and gear wear using a convolutional neural network [2]. In addition, it can also be used to predict surface wear based on surface isotropy levels [3], or in the prediction of ore crushing-plate lifetimes [4]. New technologies like machine learning (ML) [5] and deep learning (DL) [2] take analytical work to the next level. This paper presents the implementation of the machine learning approach to predict earthquakes. The used approach combines ML with proper methods of feature engineering to determine the set of statistical features that are most suitable for earthquake prediction. The main advantage of the proposed approach is that it develops our understanding of the physical aspects of the features used for modelling. Thus, the results of this work can be broadly utilized in different artificial intelligence algorithms for earthquake prediction.

An earthquake is an intense shaking of the earth’s surface due to a sudden slip of rocks in the earth’s crust, or faults near the interface between tectonic plates. The earliest earthquake with descriptive information occurred in China in 1177 B.C [6]. The earliest seismoscope was that invented by the Chinese philosopher Chang Heng in A.D. 132 [7]. Although earthquake monitoring and recording is now well developed, humans remain unable to predict a major earthquake, nor understand its exact cause. Nevertheless, earthquakes and their consequences are among the most life-changing events in the history of humanity. Seismic activities and their aftermath disasters been the cause of more human life loss than all other natural hazards combined during the past two decades [8] because many countries are located in highly seismically active zones [9,10,11].

Accurate prediction of earthquakes can potentially save lives and spare humanity further devastation. Therefore, predicting the time and magnitude of earthquakes is one of the fundamental goals of geoscience [12]. Despite long time-series of observations and field research, the accurate prediction of the size or timing of earthquakes remains a challenge [13,14]. Even more concerning is that devastating subduction earthquakes with magnitude 9.0 or more are considered unpredictable [14].

Seismology is a field in which big data is intensively generated [15]. Recent few years have demonstrated rapid growth in the application of ML and other techniques of big data analysis in seismology [16,17]. The main goals in ML, as expressed by [18], are identifying some general patterns or features in data and dimensionality reduction. Special attention has been paid to the detection of specific earthquake precursors in past studies [12,19].

There have been a number of cases that have shown successful implementation of ML in earthquake warning systems for predicting real earthquakes [20,21], their geographical coordinates [22,23] and other aspects. Combining physically based and ML-based models, a synergetic effect can be obtained when physical intuition insights are empowered by the strength of data-driven methods [15,18,24].

The recent earthquake prediction competition hosted by Los Alamos National Laboratory (LANL) in cooperation with Kaggle.com [25] attracted hundreds of data scientists globally with the goal of predicting time to failure (TTF) using acoustic data (AD) as a source of information for modelling. The current paper shares the findings from the datasets provided by organizers of the LANL earthquake prediction competition are analyzed using the training model developed for this study, and then discussed. The goal is to use seismic signals to predict the timing of laboratory earthquakes

## 2. Data and Methods

### 2.1. Data

A laboratory experiment that closely mimics real earthquakes is described in [12]. The main idea of the modelling technique is the slow relative motion of rigid, usually steel, plates pressed against each other and separated by a thin granular layer. This granular layer mimics the contact surface of the layer between tectonic plates in which rocks are located. A laboratory quake machine reproduces the stick-slip motion of conjunct plates; acoustic emission from granular gauge interlayer and contact stress values are constantly recorded against the time that remains to failure of the granular layer. These periodic failures are accompanied by a drastic increase in acoustic emission and a drop in contact stress, and are considered to be analogous to real earthquakes [26]. It is recognized that the greater the drop in stress, the more intense the ground motions during real earthquakes [27]. Because a material emits acoustic signals in the course of work and especially before failure, a similar approach may be used for predicting not only real earthquakes but also other types of failures in nature and industry, such as landslides, avalanches, and failure of machine parts [12].

For modelling purposes, a training dataset provided by the LANL Earthquake Prediction Competition hosted by Kaggle.com [25] was used. The dataset comprised a two-column csv file in which AD values derived from the acoustic signal from a laboratory machine gauge layer were recorded against TTF. The experimentation consisted of repeated cycles of model “earthquakes” (EQs). The training dataset contained the record of 16 full cycles between earthquakes. The length of the cycles varied from 7 to 16 s. The data for incomplete cycles were at the head and the tail of the training dataset. This is because the training dataset was cut from another bigger file of records. The goal was to build a model that can predict TTF for a given piece of recorded AD consisting of 150,000 entries. The length of the time window for 150 K pieces of AD was approximately 0.04 s and therefore it may be considered a single time spot.

### 2.2. Methods

**Figure 1:**
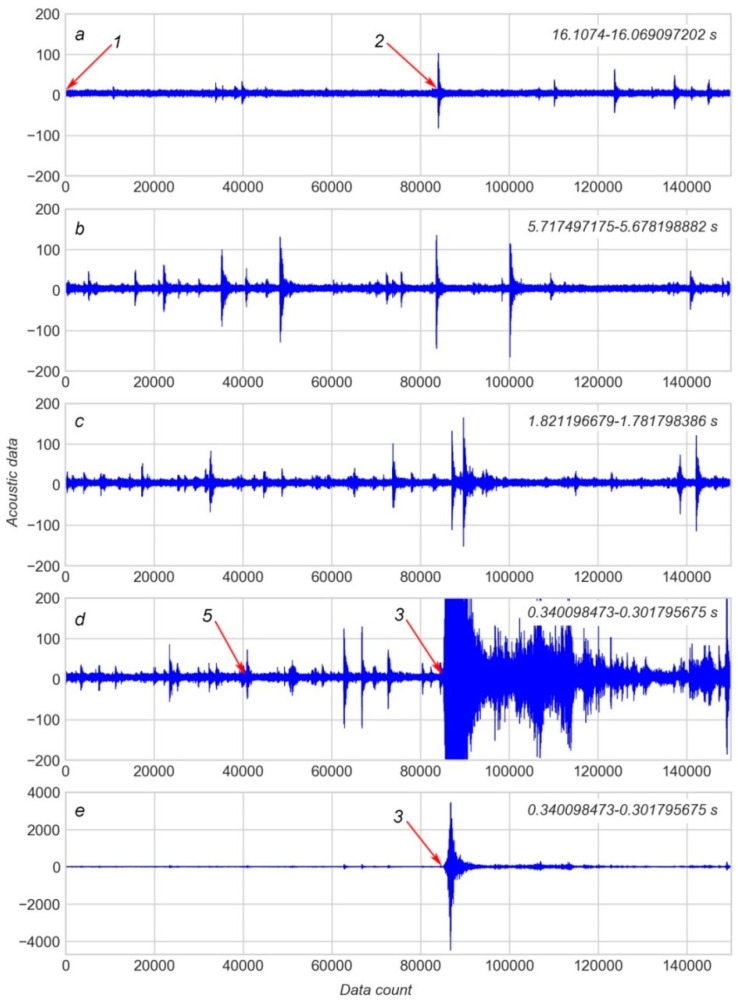
Plot of acoustic data in the 8th “earthquake” cycle from the dataset provided by [25]: (**a**)—the beginning of the seismic cycle; (**b**,**c**)—middle parts of the seismic cycle; (**d**,**e**)—part of the seismic cycle with the earthquake (EQ) event at different levels of magnification. Arrows are explained in the text.

The training dataset was split into 17 files, each containing an AD and TTF for one separate cycle. The first part of the work was carried out using a file for the longest cycle, i.e., the 8th piece in the training dataset. The first TTF in this cycle was 16.1074 s. Figure 1 shows several examples of 150 K pieces of AD for this cycle.

The beginning of the cycle is covered by the first 150 K piece (Figure 1a). Data provided in Figure 1b,c illustrates the gradual increase of spikes of AD during the seismic cycle. The piece which contains the EQ event is shown at different levels of magnification in Figure 1d,e. The role of these spikes is discussed further below. In the final stage of work, modelling was performed using all datasets provided by [25].

The XGBoost library providing the gradient boosted trees approach [28] was used for modelling. It is currently agreed that this technique leads to the best performance compared to other modelling algorithms [29]. For example, XGBoost was used to determine the dominant frequency of an eruptive tremor of the volcano Piton de la Fournaise [30]. XGBoost stands for eXtreme Gradient Boosting. This algorithm implements an ensemble of decision trees and uses gradient boosting to build models more accurate than the single decision tree or random forest approaches.

Model quality was assessed by mean absolute error (MAE) in 6-fold cross-validation (CV). Cross-Validation is used to estimate model quality by splitting the training dataset into *n*-folds. One of the folds is used for model validation, while the rest *n*−1 folds are used for modelling. Modeling is repeated *n* times, so every fold is used once as a validation dataset. MAE is one of the metrics used to estimate model accuracy. It counts the mean absolute difference between the value of the target parameter (TTF in our case), which is predicted by the model, and the actual parameter value from the validation dataset. Python 3.7 and the necessary libraries, such as pandas, sklearn, and xgboost, were employed to carry out the study.

The main aim of this study was to find an appropriate set of features derived from AD that gives the least MAE in CV. A detailed approach to feature engineering is discussed further below. Since the speed of data processing is critical for the detection and early warning of earthquakes [15] the goal of this work was to determine the feature(s) that are not only useful for building ML models with acceptable accuracy but also enable relatively rapid processing of real-time data.

## 3. Feature Engineering

The following key approach was used for feature engineering. In the first step, it is assumed that the distribution of AD is the source of useful features. This assumption is based on “common sense” suggestions, observation of changes in AD distribution over time (see Figure 1) and also on results published in related works [12,26].

It is evident that stick-slip failure (see arrow 3 on Figure 1) is preceded by a number of spikes of AD (see arrow 2 and similar symbols on Figure 1). These spikes appear as a result of micro failure events and may predict TTF [12,13]. Generally, the shorter the TTF the more frequent the AD spikes. Hence, the statistical characteristics of AD may serve as features for modelling.

As shown in [26] instantaneous statistical characteristics of AD appear as a “fingerprint” of the fault zone stress state. The variance of the seismic signal is the most important feature, although other statistical characteristics are also important [26,29,31]. The authors of [13] stressed that the kurtosis of the acoustic signal is an additional powerful feature for the prediction of TTF.

A total of 18 statistical features were derived from each of the 150 K pieces of AD in this work. Nine of these statistical features were maximum, minimum, mean, standard deviation, (standard deviation)/(mean), skewness, kurtosis, mode, number of mode appearance. The remaining nine features were percentiles at the following percent levels: 1st, 5th, 10th, 25th, 50th, 75th, 90th, 95th and 99th. The “maximum” and “minimum” features were calculated but not used for modelling because these features were only used to indicate the main EQ event due to their outstandingly large values.

**Figure 2:**
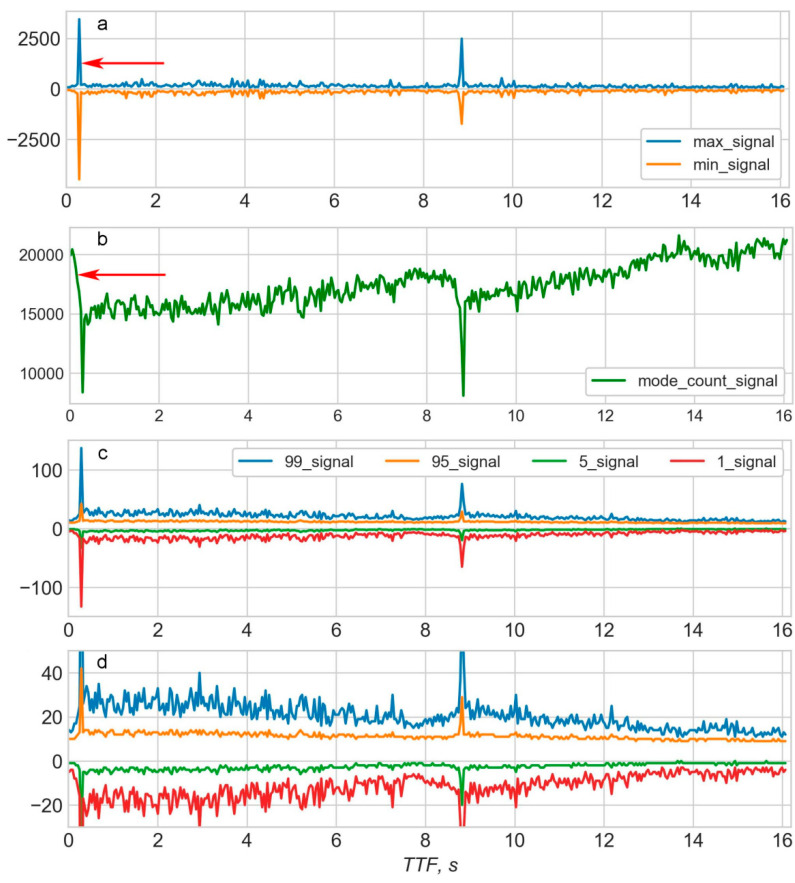
Some of the features plotted against time to failure (TTF) for the 8th “earthquake” cycle: (**a**)—maximums and minimums; (**b**)—number of mode appearance; (**c**,**d**)—99th, 95th, 5th, and 1st percentiles.

Using the initial data sequence from the 8th cycle in the LANL dataset [25], a database of statistical features was created as a result of feature calculation for every subsequent 150 K portion of AD. The database contains 413 rows which cover the TTF range of 16.0691–0.0156 s. Figure 2 shows several features plotted against TTF. It is evident that at least some of the features correlate with TTF, for example, the number of mode appearances (see Figure 2c, “mode_count_signal”). Another point to consider is that a certain portion of data is recorded after the EQ at approximately 0.3 s (arrow on Figure 2a). Values of any given feature 0.2–0.3 s after an EQ are similar to those long before the EQ in the early stage of a seismic cycle. If values recorded after the EQ were incorporated, then an additional error would appear in the model. This is due to the similarity in feature values at the beginning and the end of a cycle (arrow on Figure 2b).

In order to increase the model accuracy, all tail rows which correspond to the period after the EQ should be deleted from the database of statistical features. Another rationale for deleting data after an EQ is that the main goal of modelling using data from laboratory EQs is to identify features that would be useful to predict real EQs. It is obvious that in reality only data before an EQ would be used for prediction. Any data after an EQ has neither a logical nor practical sense for prediction of that particular EQ.

Due to the development of modelling tools such as Python and appropriate libraries, training a model can be performed rapidly using only several lines of code. The major challenge in training any model is determining which features should be used.

**Figure 3:**
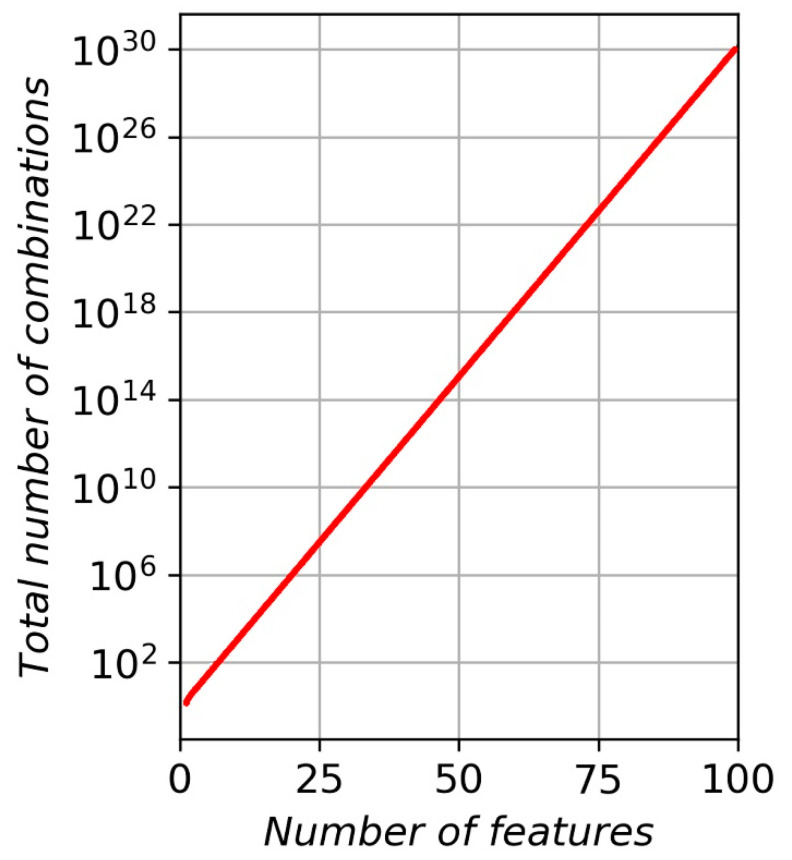
The total number of possible combinations versus the number of features.

In the current work, the final selection of features was based on the building of different models to compare MAEs and picking the best combination of features that gives the lowest MAE. However, according to the well-known curse of dimensionality, the total number of possible combinations of features increases far faster than the number of features in the set (Figure 3).

For example, four features in the set give fifteen possible combinations, 7–127, 10–1023, 15–8191, 16–64,995, 18–262,143, and so on. The “brute force” (BF) method of feature engineering involves sequential modelling and CV score calculation for each combination of features and picking the combination with the least MAE. This method guarantees that the best combination of features is determined. However, BF is time consuming if the number of features exceeds some threshold. In general, the higher the number of features analyzed, the greater the time required to solve the model. For example, a set of 43 features [13] gives a total of 8.796 × 10^12^ combinations, which would require a significant amount of time to find the best combination.

In the paper [13], only two best features were chosen from 43 for prediction of TTF. This means that most of the features are either excessive or not suitable for modelling. Therefore, it can be concluded that the first step in feature engineering is to exclude all non-significant features from the set. Every feature excluded can significantly decrease the total amount of combinations to examine during the BF approach. In our, case excluding only two features decrease the number of combinations from 262,143 to 64,995 (16 features instead of 18).

The “maximum” and “minimum” features can be excluded based on the following reasoning: The maximum values of AD in 150 K pieces is equivalent to the 100th percentile value. This study uses the “99th percentile” feature which is close to the 100th percentile; therefore the 100th percentile (i.e., “maximum”) is superfluous and can be excluded. Similarly, the “minimum” feature is equivalent to the “0 percentile” and can be excluded as the “1st percentile” feature has already been considered. The only reason for calculating “maximum” and “minimum” features is because they are needed for correct identification of the 150 K piece which contains the EQ. It also helps to correctly delete tail rows containing AD after the EQ. After excluding “maximum” and “minimum” features, 16 features remain in the model, giving a total of 64,995 possible combinations.

The backward feature elimination technique (BFE) was employed for reducing the number of features. The rationale behind using this method in the current study is that, if there is a total of *n* features in a set, then there are *n* possible combinations of (*n*−1) features in the subset. Assuming that the vast majority of features are either bad or neutral for model quality, it is highly probable that the MAE for the model—which uses all *n* features—would be bigger than the least MAE for *n* models using (*n*−1) features. If so, then only one model is needed such that it uses all *n* features, and MAE*_n_* is then calculated; thereafter, *n* models are required, each of which uses one of the possible subsets of (*n*−1) features. It is also important to choose the subset which results in the least MAE*_n_*_−1_. If a full set of *n* features contains at least one feature that is bad or excessive for the model, then MAE*_n_* would be greater than or equal to the least MAE*_n_*_−1_. This bad or excessive feature should be absent in the subset that generates the model with the least MAE*_n_*_−1_. Thus, this feature can be excluded from the set of features. BFE takes about a minute in semiautomatic mode to exclude one feature and can be fully automated if necessary. BFE is consistently used to reduce the number of features from *n* to about 10. Thereafter, the straight BF method is used to find the combination of features that gives a model with the least MAE.

The next important point to consider is how many CV cycles are necessary for every step of the work. Each single CV cycle returns the mean MAE for only six calculations in total. Therefore, the resulting MAE varies at the second decimal point from one run to the other. In order to decrease the variance of MAE the number of repetitions (cycles) of CV should be increased. Two cycles (CV-2) were used for BFE and 500 cycles (CV-500) were used in the final calculation of MAE for the best combination of features. Using CV-500 enables MAEs that are stable to the third decimal point to be obtained.

Features other than AD may also be useful for TTF prediction. It may be seen that sudden spikes in the signal are presented in the AD–TTF diagram (see Figure 1a–d). The lower the TTF, the more often spikes occur. As stated in [12], these spikes appear due to micro shifts in the gauge layer.

**Figure 4:**
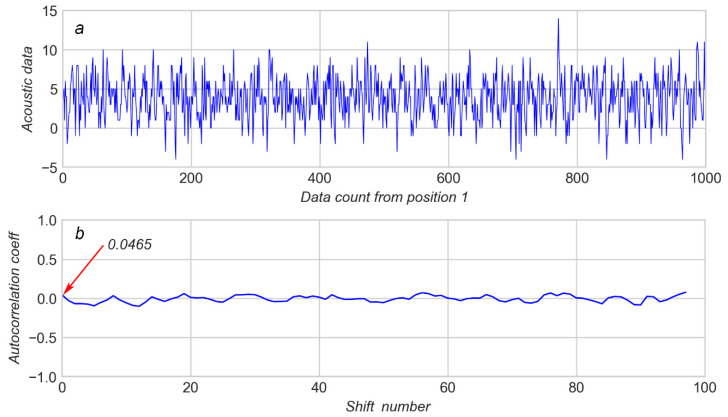
(**a**): The 1000 K window of acoustic data from arrow 1 (see Figure 1); (**b**): corresponding autocorrelation coefficients.

**Figure 5:**
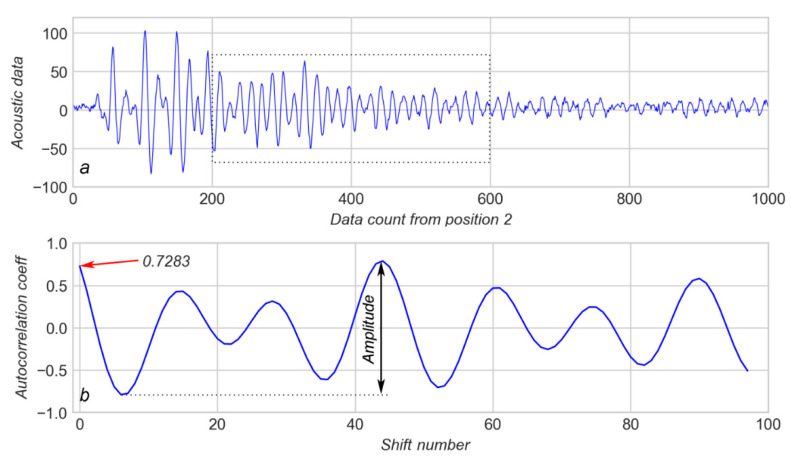
(**a**)—1000 K window of acoustic data from arrow 2 (see Figure 1); (**b**)—corresponding autocorrelation coefficients.

Figure 4a shows a short portion of 1000 values for the AD–TTF diagram corresponding to arrow 1 on Figure 1a. This portion of data contains no spikes in the AD and the AD distribution seems to be random. Figure 5a shows a short portion of 1000 values for the AD–TTF diagram corresponding to arrow 2 (see Figure 1a); that is, the beginning of the first significant spike observed in the AD. The AD distribution, in this case, seems to be more or less periodic with a gradual increase of random constituents. Spikes in AD are characterized not only by an increase in AD amplitude but also by the grade of AD periodicity.

**Figure 6:**
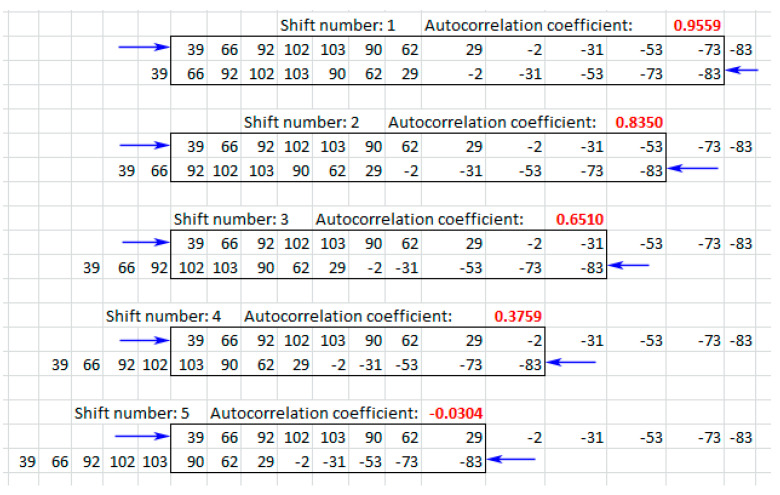
Several first calculations of autocorrelation coefficient (AC) for 13 consequent acoustic data (AD) values corresponding to arrow 2 in Figure 1.

This grade of AD periodicity may be assessed by the autocorrelation coefficient (AC). Figure 6 shows several first steps for calculating the AC for 13 consequent AD values corresponding to arrow 2 in Figure 1a. These values are {39,66,92,102,103,90,62,29,−2,−31,−53,−73,−83}. Each AC value (red numbers in Figure 6) is calculated for a given sequence of AD that is duplicated against itself and shifted by one position. For example, after the first shift there are two sequences of 12 numbers that overlap: {39,66,92,102,103,90,62,29,−2,−31,−53,−73} and {66,92,102,103,90,62,29,−2,−31,−53,−73,−83}. These overlapping sequences are included in a black rectangle under the words “Shift number: 1”. Calculating the usual correlation coefficient for this pair of sequences gives 0.9559. This is the first value of AC. After the second shift, only 11 numbers remain in each overlapped sequence (see black rectangle under “Shift number: 2”). This pair of sequences gives a correlation coefficient of 0.8350. This is the second value of AC. Further shifts give new values of AC. Figure 6 shows five consequent shifts, but the number of shifts may be arbitrary. The only limitation is the length of the initial sequence. In our case we use a sliding window of 1000 AD values, therefore quite a large number of shifts may be used.

A total of 98 shifts were used to calculate the ACs for every position of the “sliding window”, containing 1000 values of AD. The results from the calculation of the AC for AD in Figure 4a and Figure 5a are shown on Figure 4b and Figure 5b respectively.

It can be observed that a highly aperiodic AD (see Figure 4a) produces an AC which varies in a very narrow range of about ±0.1 (Figure 4b). In contrast, a highly periodic AD (Figure 5a) produces an AC which varies in a wide range of about ±0.8 (Figure 5b). If AD is aperiodic it means that no spikes are present in AD inside the sliding window. The greater the AC amplitude (see Figure 5b), the more periodic AD. Therefore, high values of AC amplitude mean that the sliding window contains spikes of AD.

An additional sign of AD periodicity is the first value of AC (see arrows on Figure 4b and Figure 5b). The more periodic AD, the higher the first value of AC.

**Figure 7:**
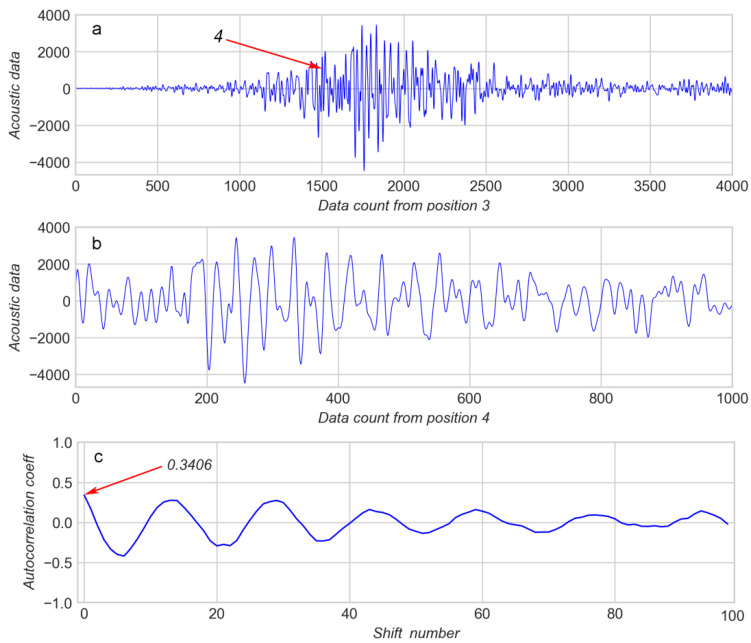
(**a**): “earthquake” event, see arrow 3 on Figure 1; (**b**): 1000 K window of acoustic data from arrow 4; (**c**): corresponding autocorrelation coefficients.

These observations can be checked on AD for an “earthquake” (see arrow 3 on Figure 1d,e and Figure 7). Figure 7a represents AD for a whole EQ event, and Figure 7b contains AD for a sliding window starting at the position indicated by arrow 4. It can be noted that AD in Figure 7b are less periodic than those in Figure 5a. In accordance with this difference in periodicity, the first value of AC in Figure 7c (0.3406) is less than that in Figure 5b (0.7283). The amplitude of AC in Figure 7c is also less than that in Figure 5b.

**Figure 8:**
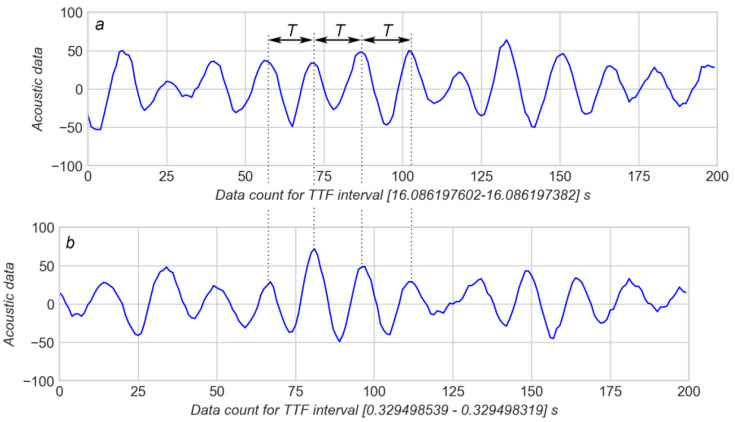
Comparison of frequency of AD splashes far from (**a**) and near (**b**) the “earthquake”: (**a**)—first 200 AD values in the dashed window in Figure 5a; (**b**)—first 200 AD values from arrow 5 in Figure 1.

The frequency of AD oscillation was considered during a spike as an additional feature which can be used for modelling. However, the comparison of AD of early and late spikes (see arrows 2 and 5 in Figure 1) shows that periods of oscillation *T* for both cases are approximately equal (Figure 8). Therefore, the frequency of AD oscillation during a spike was not used for modelling.

This way, three major parameters were used for modelling which are: acoustic data (AD); the first value of AC on every “sliding window” (AC_first); the amplitude of AC on every “sliding window” (AC_ampl). Each 150 K piece of AD contains 150 sliding windows and, therefore, 150 values of AC_first and 150 values of AC_ampl. Because 16 statistics were calculated for each of three parameters, the overall number of features considered was 48.

These features were calculated for every separate seismic cycle in the database provided by [25]. For every portion containing 150 K of AD, the features were calculated and recorded with the corresponding TTF in the separate file of features. Since the TTF change during 150 K of AD was just 0.04 s, the TTF value is considered a constant during any given 150 K piece. The last value of TTF in the 150 K piece was used as this constant time. Maximum and minimum values of AD were also recorded; they allowed the row that contained the EQ event to be located. All rows after the row with EQ event were deleted according to the reasons explained above. It should be noted that in all files TTF for the EQ event was approximately the same (near 0.3 s).

Finally, all separate files with features were merged into one database.

## 4. Results and Discussion

**Figure 9:**
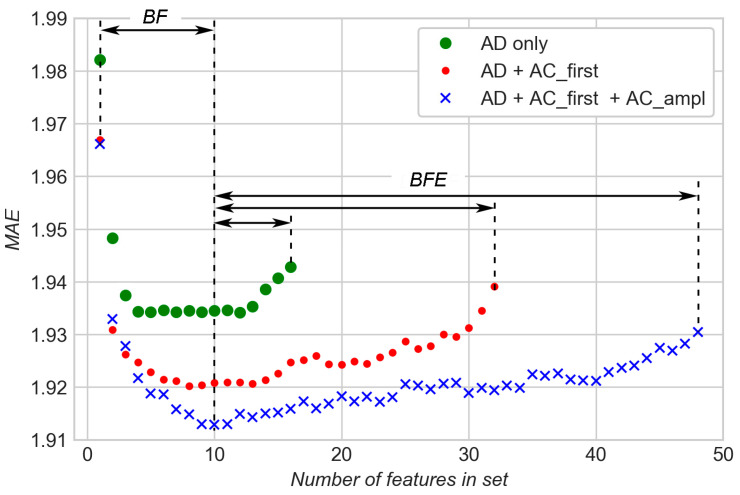
Comparison of modelling results for a different combination of features.

Figure 9 shows the best MAE obtained for any given number of features in the subset of features. Three sequences of dots represent three sets of features: 16 features for AD only; 32 features for AD+AC_first; and 48 features for AD+AC_first+AC_ampl. Each sequence of dots is a function “MAE vs. Number of features” for the corresponding set of features.

It is evident that in each of the three sets of features there are good, excessive (neutral), and bad features. During BFE, bad features were gradually removed from subsets of features and “MAE vs. Number of features” functions slowly decreased for each of three sets with the decreasing number of features. As the number of features reached 10, the BF method was used to find the best combination of features that gave the least MAE. It can be seen that MAE decreases significantly for each of the three functions in the early stages of the increasing number of features during the BF stage. This means that useful features are gradually added to the subsets. After a certain number of features, MAE stabilizes and minimums are reached. Addition of more features does not lead to a decrease of MAE, so these additional features are excessive (neutral) for modelling.

The result shows that all three parameters—AD, AC_first, and AC_ampl—have potential for predicting TTF. Even statistical features which are derived solely from AD result in MAE at the level of 1.93 s if chosen in the optimal combination. Addition of AC_first and AC_ampl gradually reduces levels of the best MAEs to 1.92 and 1.91 s, respectively.

Table 1 shows minimal MAEs for three subsets and the corresponding features in each subset. According to Table 1, the only features that appear in all three optimal sets are “mean of AD” and “number of mode appearance of AD”. Hence these features possess the strongest predictive ability; however, models built solely using these two features are not as good as those with optimal combinations. This means that some synergistic effect appears when other features are added to the models, which leads to a decrease of MAE.

It should be noted that optimal sets of features may vary if the training dataset changes. Therefore, the value of this work is not in these particular optimal sets of features. The main outcome here is that autocorrelation is a valuable parameter which has a significant influence on model quality.

It should also be noted that the calculation time for AC_first is at least one order of magnitude less than that for AC_ampl. Calculation of AC_first requires just one shift (see Figure 4), whereas the calculation of AC_ampl requires 98 shifts. Since processing time can be critical in some cases [32], the usefulness of AC_ampl may be questionable from a practical point of view.

According to the results obtained and presented above, the calculation of AD autocorrelation in a given sliding window is a useful operation that helps to determine how the periodicity of AD. Generally, the more periodic the AD, the shorter the TTF. This leads to the idea of including autocorrelation calculation as a core operation of the deep learning algorithm instead of or in conjunction with the usual convolutional operation. As shown in [33], deep learning modelling can be adapted to recognize tectonic tremors. One of the possible directions of future research may be incorporating autocorrelation of acoustic (tectonic) data into existing algorithms of artificial intelligence in the field of seismology.

## 5. Conclusions

Periodic spikes of acoustic data is a phenomenon that can be used on its own to determine TTF during laboratory earthquake tests. Only certain combinations of statistical characteristics lead to a model with optimal performance.

The backward feature elimination approach in combination with the brute force method can be successfully used to find this optimal combination even for relatively big sets of candidate features. The backward feature elimination stage allows the number of features to be significantly reduced; therefore, subsequent brute force attempts can be used to select the combination of features that yields the model with the least MAE.

Three major parameters useful for predicting TTF were determined as follows: distribution of acoustic data, the first values of autocorrelation coefficients in 1000 K sliding windows, and the amplitudes of these autocorrelation coefficients. A total of 48 statistical features were derived from these three parameters. The best combination of statistical features allowed a model with a mean absolute error of 1.913 s to be obtained.

The autocorrelation of acoustic data series is an important parameter. It provides additional information about the grade of acoustic data periodicity. The greater the amplitude and the first value of the autocorrelation coefficient sequence, the more periodic the acoustic data. High periodicity means that spikes of acoustic data are present that serve as the precursors of laboratory earthquakes. Calculation of autocorrelation coefficients can be used as a valuable operation in artificial intelligence systems deployed for real earthquake prediction.

## Figures and Tables

**Table 1:** Optimal subsets of features and corresponding mean absolute errors (MAEs).

Statistic Feature	AD	AD+AC_First		AD+AC_First+AC_Ampl		
		AD	AC_First	AD	AC_First	AC_Ampl
mean	+	+		+		
standard deviation						+
(standard deviation)/(mean)			+			
skewness						
kurtosis						
mode						
number of mode appearance	+	+		+	+	+
percentiles:						
1st	+	+				
5th				+		
10th						
25th			+		+	
50th		+				
75th	+	+		+		
90th				+		
95th			+		+	
99th						
MAE (CV-500)	1.9343	1.9210		1.9130		

## Nomenclature

AC	Autocorrelation Coefficient
AC_ampl	The amplitude of AC on sliding window
AC_first	The first value of AC on sliding window
AD	Acoustic Data
BF	Brute Force method
BFE	Backward Feature Elimination
CV	Cross-Validation Algorithm
EQ	Earthquake
MAE	Mean Absolute Error
ML	Machine Learning
TTF	Time To Failure
